# *Otx2* Gene Deletion in Adult Mouse Retina Induces Rapid RPE Dystrophy and Slow Photoreceptor Degeneration

**DOI:** 10.1371/journal.pone.0011673

**Published:** 2010-07-21

**Authors:** Francis Béby, Michael Housset, Nicolas Fossat, Coralie Le Greneur, Frédéric Flamant, Pierre Godement, Thomas Lamonerie

**Affiliations:** 1 Institut de Génomique Fonctionnelle de Lyon, Centre National de la Recherche Scientifique, Ecole Normale Supérieure de Lyon, Institut National de la Recherche Agronomique, Université de Lyon, Lyon, France; 2 Institut de Biologie du Développement et Cancer, Centre National de la Recherche Scientifique, Université de Nice Sophia-Antipolis, Nice, France; 3 Embryology Unit, Children's Medical Research Institute, Sydney Medical School, University of Sydney, Wentworthville, Australia; University of Washington, United States of America

## Abstract

**Background:**

Many developmental genes are still active in specific tissues after development is completed. This is the case for the homeobox gene *Otx2*, an essential actor of forebrain and head development. In adult mouse, *Otx2* is strongly expressed in the retina. Mutations of this gene in humans have been linked to severe ocular malformation and retinal diseases. It is, therefore, important to explore its post-developmental functions. In the mature retina, *Otx2* is expressed in three cell types: bipolar and photoreceptor cells that belong to the neural retina and retinal pigment epithelium (RPE), a neighbour structure that forms a tightly interdependent functional unit together with photoreceptor cells.

**Methodology/Principal Findings:**

Conditional self-knockout was used to address the late functions of *Otx2* gene in adult mice. This strategy is based on the combination of a knock-in *CreERT2* allele and a *floxed* allele at the *Otx2* locus. Time-controlled injection of tamoxifen activates the recombinase only in *Otx2* expressing cells, resulting in selective ablation of the gene in its entire domain of expression. In the adult retina, loss of Otx2 protein causes slow degeneration of photoreceptor cells. By contrast, dramatic changes of RPE activity rapidly occur, which may represent a primary cause of photoreceptor disease.

**Conclusions:**

Our novel mouse model uncovers new Otx2 functions in adult retina. We show that this transcription factor is necessary for long-term maintenance of photoreceptors, likely through the control of specific activities of the RPE.

## Introduction

The molecular basis of some forms of *retinitis pigmentosa* [1] and of late onset retinal degeneration, in particular age-related macular degeneration (AMD) remains obscure. Studying these diseases requires animal models designed to explore gene function in adults. Unfortunately, such tools are still largely unavailable [2]. Knockout models often result in deficiencies that occur during development rather than in mature retina, precluding assessment of gene function in adults.

One transcription factor that is expressed in retinal cell types (photoreceptors and retinal pigment epithelium), where abnormal function can lead to various diseases, is the homeoprotein Otx2. Otx2 is well studied for its role in embryonic head formation [3], and differentiation of various types of neurons [4], [5]. It has also been proposed to modulate the plasticity of the visual cortex through a paracrine mechanism [6]. Recent studies have demonstrated its requirement at many steps of eye development. Otx2 first acts at mid-gestation allowing the differentiation of the RPE territory [7], [8]. It is then necessary for neural retina development, maturation and photoreceptor fate determination [9], and for bipolar cell terminal differentiation after birth [10], [11]. These studies have proven very informative for the role of *Otx2* in development of the retina. Still, little is known about its function in the adult, although it is strongly expressed in the adult retina [10], [12], [13].

Some patients that suffer from microphthalmia and anophthalmia have been reported to carry *OTX2* mutations [14]. It was also noted that two eye diseases that are likely unrelated to development, *retinitis pigmentosa* (RP) and Leber congenital amaurosis (LCA) sometimes developed in these patients. It has also recently been reported that *Otx2*, and *Crx*, are highly expressed in retinoblastoma tumor cells in patients [13]. The *Otx2* gene uses three different promoters and has many enhancers that act at different times and cellular types spread over 300 kb [15], [16], [17]. Therefore it is quite possible that changes in noncoding sequences regulating *Otx2* transcription might lead to retinal disease. To explore the functions of Otx2 in the adult eye, we took advantage of the novel genetic approach coined self-knockout that we previously used to study *Otx2* early functions [18] and which allows time-controlled and very efficient knockout of Otx2 only in cells that express it.

## Results

### Evaluation of *Otx2* knockout in the adult retina

**Figure 1:**
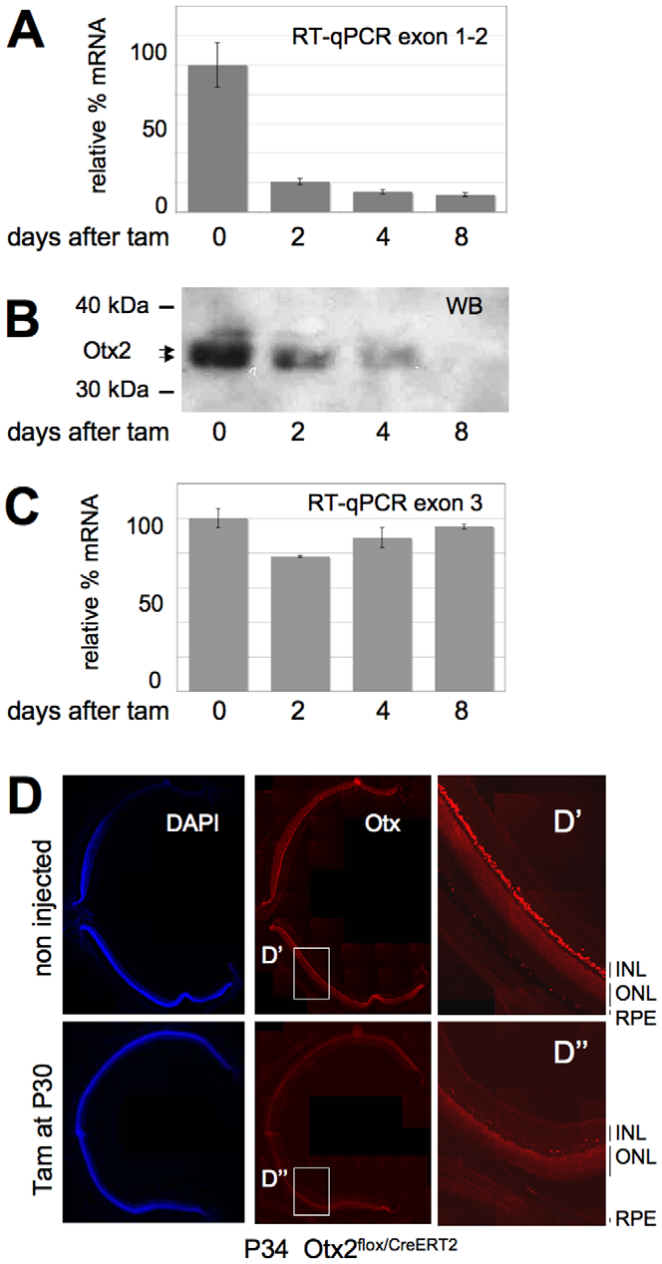
Efficient loss of *Otx2* gene products after self-knockout in adult retina. **A–C.** Time course analysis of *Otx2* gene products following tamoxifen injection at P30 in *Otx2^flox/CreERT2^* mice. Relative levels of full-length (A) and total (normal + deleted) (C) *Otx2* mRNA measured by RT-qPCR are shown. Corresponding levels of Otx2 protein (B) is shown in western blots. Position of size markers and Otx2 protein are indicated. Error bars in A and C are standard deviation. **D.** Staining of nuclei (DAPI) and Otx proteins on identical retina sections from control and mutant mice of the same genotype 4 days after tamoxifen injection. Shown are stitching of overlapping fields reconstituting a whole section. Right panel: magnification of boxed D′ and D″ areas. RPE: retinal pigment epithelium; ONL: outer nuclear layer; INL: inner nuclear layer.

Sustained expression in RPE, photoreceptors and bipolar cells (BC) (Fig. S1) suggested that *Otx2* gene could exert functions in the mature retina. In order to address this issue, we used *Otx2^flox/CreERT2^* adult mice (Fig. S2). In this model, tamoxifen inducible CreERT2 recombinase is expressed from one *Otx2* allele whereas the other allele carries two *loxP* sites flanking the homeodomain coding exon 2. The *floxed* allele expresses normal levels of Otx2 protein. As the *CreER^T2^* gene is inserted at the *Otx2* locus, tamoxifen treatment induces CreER^T2^-mediated deletion of the functional allele only in cells that express *Otx2*, a strategy we termed self-knockout [18]. We triggered *Otx2* self-knockout in P30 *Otx2^flox/CreERT2^* mice. Gene ablation in the whole retina was assessed by measurement of mRNA and protein abundance during 8 days following tamoxifen treatment. Quantitative RT-PCR (RT-qPCR) analysis of full length *Otx2* transcript revealed a reduction of 90 to 95% of its abundance after 2 days. This level remained stable at later times (Fig. 1A). Western blot analysis also showed a decrease of Otx2 protein levels (Fig. 1B), which became undetectable 8 days after tamoxifen injection. Consistently, in histological sections, *Otx2* expression in the RPE, photoreceptor layer and inner nuclear layer appeared dramatically reduced throughout the retina 4 days following tamoxifen treatment (Fig. 1D). However, transcription of the *Otx2* locus after self-knockout was not affected: quantification of deleted and full length mRNAs by RT-qPCR using exon 3 primers (Fig. 1C) and *Cre* mRNA by *in situ* hybridization (Fig. S3, right panel) revealed a steady-state level of mRNA, ruling out *Otx2* autoregulation in the mature retina. Together, these results show that triggering self-knockout in adult retina leads to an almost complete Otx2 protein loss within 8 days.

### Alterations of photoreceptors

**Figure 2:**
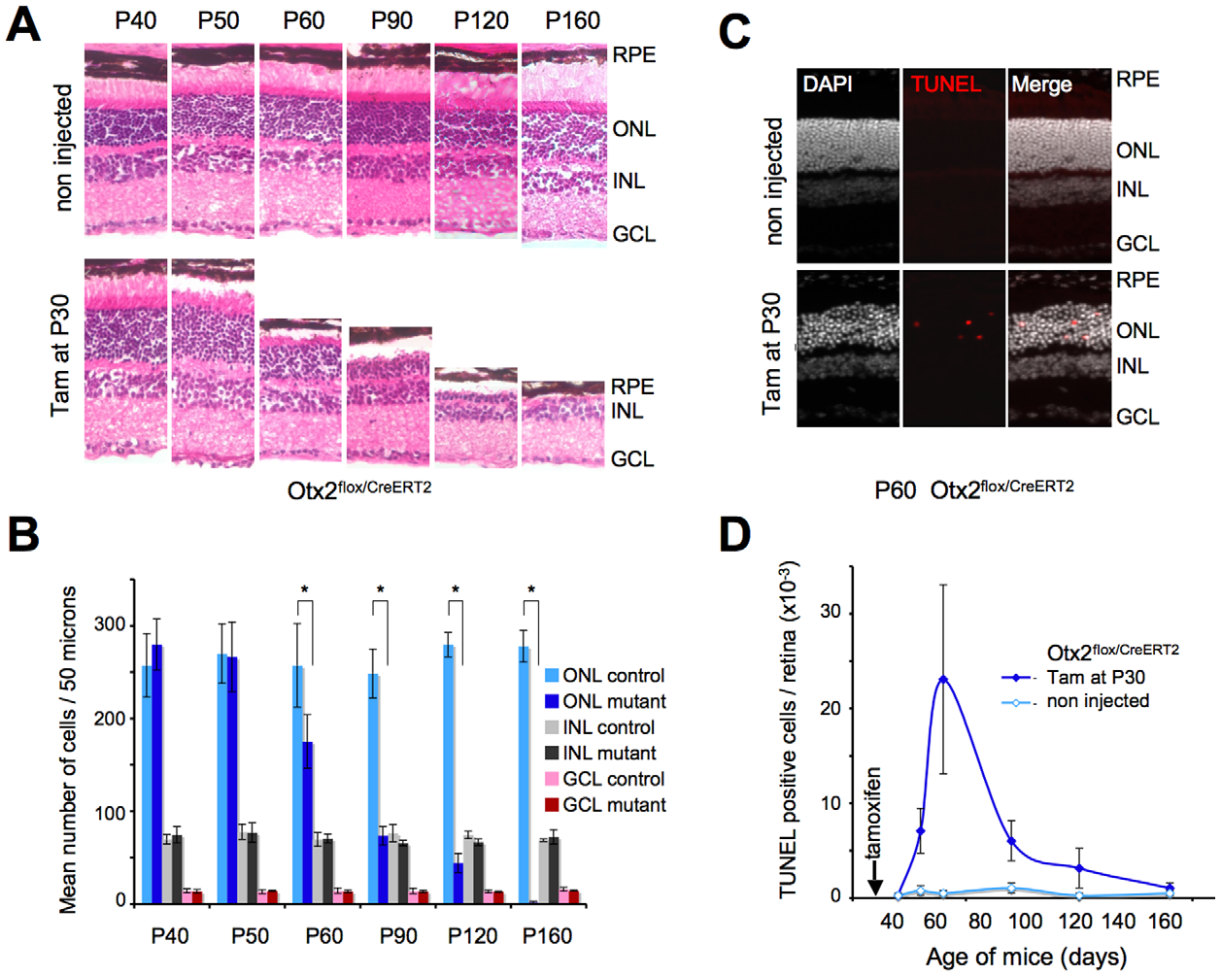
Loss of Otx2 leads to photoreceptor cell degeneration. **A.** Histology of control (non injected) and mutant (Tamoxifen administrated at P30) series of *Otx2^flox/CreERT2^* retinas of the indicated ages. Sections are stained with Eosin and Haematoxylin. RPE: retinal pigment epithelium; ONL: outer nuclear layer; INL: inner nuclear layer; GCL: ganglion cell layer. **B.** Cell counts in the three layers retinal sections of control or tamoxifen treated (mutant) *Otx2^flox/CreERT2^* mice at indicated ages. Normalized fields of the same eye area were used. Mean cell number and standard deviation are indicated for each condition (*P<0.001). **C.** Detection of apoptotic cells in control or tamoxifen treated *Otx2^flox/CreERT2^* retinas 30 days after treatment. Left, middle and right panels show respectively DAPI staining, TUNEL labelled cells and superimposition of both images. **D.** Kinetics of apoptosis following *Otx2* gene ablation. TUNEL labelled cells were counted on normalized sections of three independent mice for each stage, each corresponding to 0.063 mm^2^ of retinal area. Error bars are standard deviation.

The consequences of *Otx2* gene ablation in adult retina were examined in a series of mice, from P40 to P160, corresponding to 10–130 days after treatment (Fig. 2A). In *Otx2^flox/CreERT2^* retina treated with tamoxifen at P30, we observed first a shortening of the photoreceptor outer segments, which were detached from the RPE from about P50. With time, the outer and inner segments progressively disappeared until by P120 they were altogether entirely absent. The whole retina was affected. In parallel we observed a progressive thinning of the outer nuclear layer, which started from around P60, until, by P160 no or extremely few photoreceptors were left. We also noted that with time from the injection of tamoxifen, the retina was increasingly detached from the RPE – although this likely was exacerbated by the histological procedure. Control *Otx2^flox/CreERT2^* animals exhibited normal retinal histology. Retinas of tamoxifen-injected *Otx2^flox/flox^* and *Otx2^+/CreERT2^* mice appeared similar to control retinas (Fig. S4) ruling out any potential nonspecific toxicity of tamoxifen treatment. Cell counting revealed progressive disappearance of photoreceptors and no change of cell numbers in both inner and ganglion cell layers (Fig. 2B). The kinetics of photoreceptor loss was identical in central and peripheral retina as well as in dorsal and ventral retina.

To investigate more closely the reason and time-course of photoreceptor disappearance we did TUNEL staining on a series of retinas from the same ages. Positive TUNEL cells could only be found in the photoreceptor layer (Fig. 2C). We studied the kinetics of apoptosis by quantifying TUNEL staining at various timepoints following tamoxifen injection. Retinas from treated mice had few apoptotic photoreceptors at P40, 10 days following tamoxifen injection. However the numbers of dying photoreceptors increased very much by P50, reaching a maximum around P60 (30 days after injection), and then slowly decreasing until P160, by which time virtually no photoreceptors remained (Fig. 2D). This kinetics was consistent with the observed decrease of thickness of the outer nuclear layer. In control mice there were very few apoptotic cells and the thickness of the outer nuclear layer remained unchanged throughout this period. Thus, Otx2 depletion in adult retina leads to selective and slow photoreceptor cell death by apoptosis.

**Figure 3:**
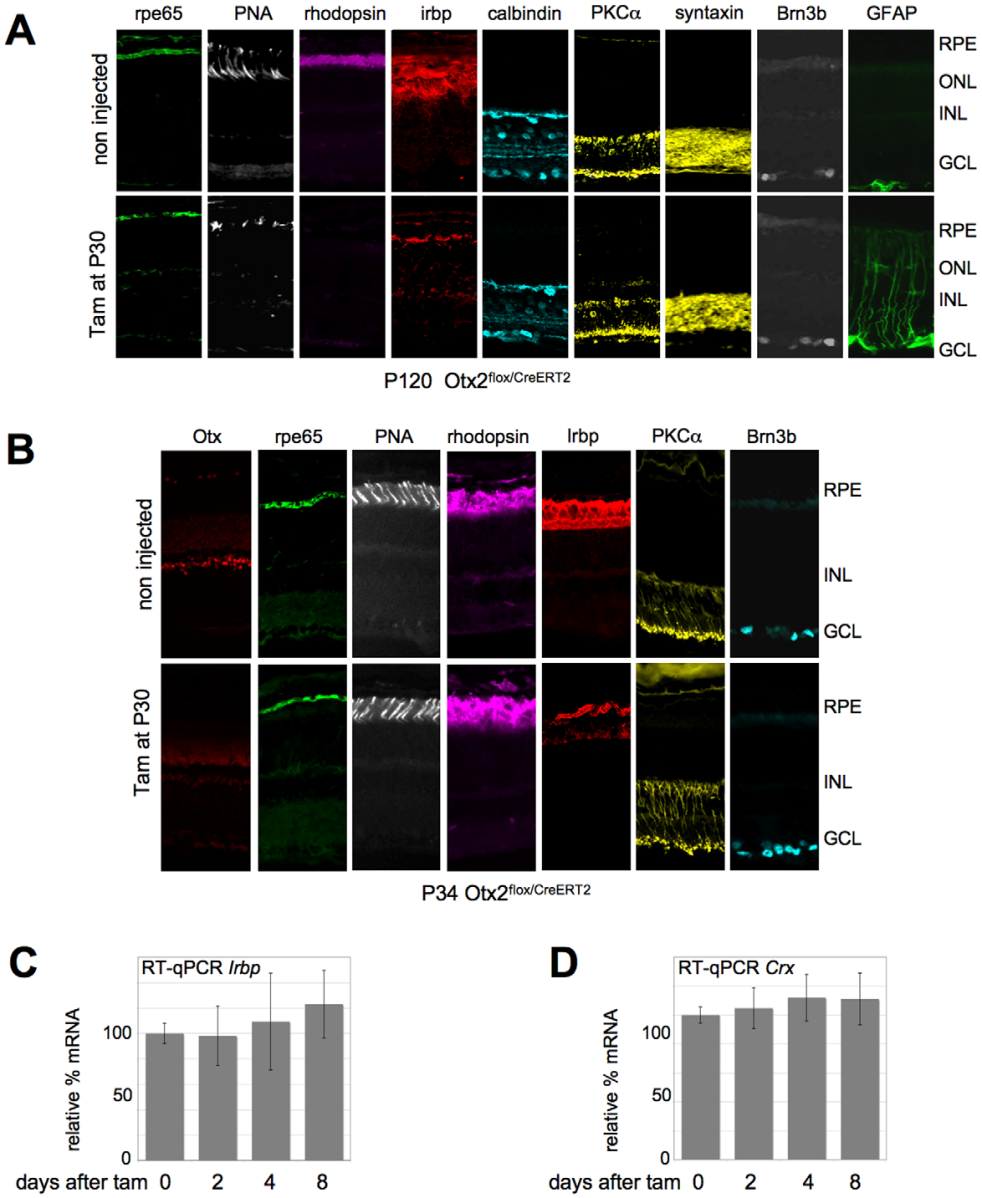
Consequences of *Otx2* loss in photoreceptor cells. **A.** Expression of cell type specific markers in P120 control (upper panels) and knockout (lower panels) retinas, following tamoxifen treatment at P30. **B.** Expression of cell type specific markers in P34 control (upper panels) and self-knockout (lower panels) retinas, following tamoxifen treatment at P30. **C.** Relative levels of *Irbp* mRNA in *Otx2^flox/CreERT2^* retina following tamoxifen treatment. **D**. Relative levels of *Crx* mRNA in *Otx2^flox/CreERT2^* retina following tamoxifen treatment. Error bars are standard deviation.

Consistent with photoreceptor alteration, PNA labelling of cone outer segments was reduced in mutants, which also showed absence of rhodopsin at P120 (Fig. 3A). IRBP, a glycoprotein of the inter-photoreceptor matrix synthesized by photoreceptors, was less abundant in mutants. We also investigated expression of cell-type specific markers in P120 mutant retinas. Syntaxin labelling indicated no expansion of the amacrine population. Expression of markers of RPE (rpe65), horizontal (calbindin), bipolar (PKC-alpha, calbindin) and ganglion cells (Brn3b) also did not appreciably change in mutant retina. However, GFAP labelling of Müller cells was enhanced in mutant retinas, a hallmark of glial reaction typical of degenerating neural tissue [19].

To find out if expression of some of the above markers was directly linked to that of *Otx2*, we analyzed their expression soon after tamoxifen injection. Four days after treatment, although Otx2 protein was strongly reduced, we observed no change in expression of most tested markers including rhodopsin (Fig 3B), with one exception, IRBP whose staining appeared decreased. This prompted us to analyze *Irbp* gene expression. Although *in vitro* experiments have suggested it to be regulated by Otx2 [20], we found no change in *Irbp* mRNA level by RT-qPCR (Fig. 3C), suggesting that its decrease in sections was either a secondary consequence of other alterations, or that it was not due to transcriptional regulation. *Crx*, a major determinant of photoreceptor gene expression, appeared another interesting candidate [21]. Otx2 protein is required during embryogenesis to activate *Crx* in photoreceptor precursors [9] and this might also be needed during postnatal development [10]. However, we failed to observe any change in *Crx* mRNA level either by *in situ* hybridization 30 days after tamoxifen administration (Fig. S5) or by RT-qPCR immediately following tamoxifen treatment (Fig. 3D). A panel of photoreceptor specific genes – *Gnat2, Rhodopsin, Grk1, Opsin1, Pde6b* - was also tested by RT-qPCR. None displayed any change over 8 days following knockout (6) showing no dramatic functional alteration of the photoreceptor itself upon *Otx2* gene ablation.

To summarize, these observations suggest that knockout of *Otx2* gene induces a specific loss of photoreceptors, which starts with a lag of about 2 weeks following the knockout and then continues during about 4 months, until no photoreceptors remain. Our observations also show a progressive shortening and degeneration of the outer segments of photoreceptors during this period. Other retinal cell types are not affected to any comparable degree.

### Functional alteration of retinal pigment epithelium

**Figure 4:**
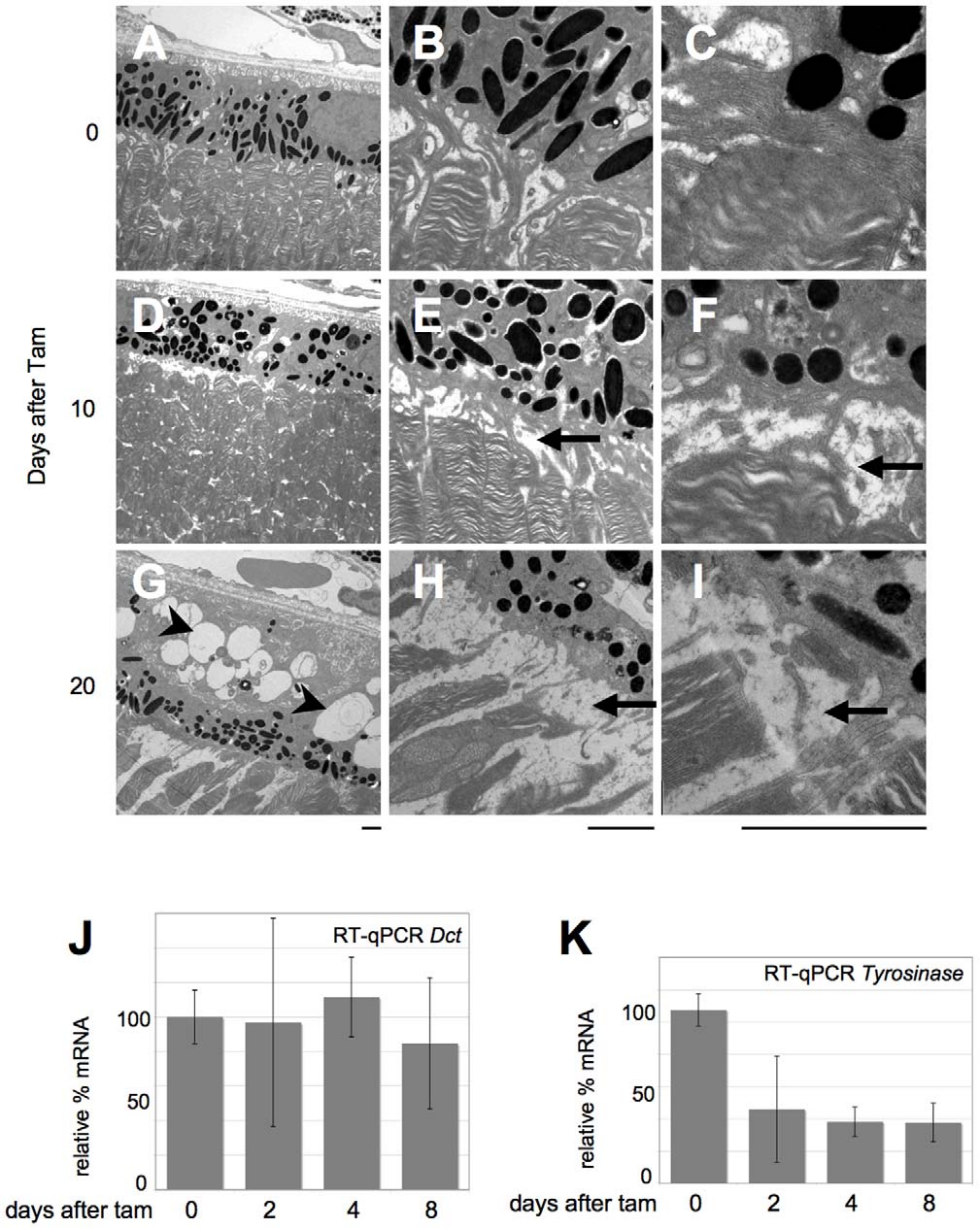
Rapid induction of RPE disease by *Otx2* self knockout. **A–I.** Transmission electron microscopy images of control (A–C) and self-knockout *Otx2^flox/CreERT2^* retina 10 days (D–F) and 20 days (G–I) after tamoxifen injection. Low (A,D,G), medium (B,E,H) and high (C,F,I) power views are presented, focusing on the area of interaction between RPE and photoreceptor disk-containing outer segments. Scale bars: 2 µm. **J–K.** Relative levels of *Dct* (J) and *tyrosinase* (K) mRNA in *Otx2^flox/CreERT2^* retina following tamoxifen treatment. Error bars are standard deviation.

The retinal pigment epithelium expresses *Otx2* at a high level, and is necessary for function and maintenance of the photoreceptors. To investigate morphological consequences of *Otx2* knockout, both on RPE cells and on photoreceptor outer segments, we performed electron microscopic analysis of control and mutant retina 10 and 20 days post tamoxifen treatment. We found rapid, progressive and severe alterations of RPE cells. Ten days after knockout, RPE cells already appeared thinner with smaller and less numerous melanosomes (Fig. 4D–F). In parallel, we found disruption of RPE contacts with photoreceptor outer segments, which resulted in widening of the inter-photoreceptor matrix space (Fig. 4E, F). This worsened in retinas 20 days post treatment, with an almost complete loss of RPE contacts with disc-containing photoreceptor outer segments (Fig. 4G–I). In addition, the melanosome and melanin content of RPE cells appeared reduced, suggesting a block in biogenesis or maintenance of this cell compartment. Low-power views also revealed two important RPE modifications: i) relocalization of the remaining melanosomes. Whereas evenly dispersed in control RPE cells (Fig. 4A), these accumulated at the apical cell side 20 days post treatment (Fig. 4G). ii) extensive vacuolization of RPE cells (Fig. 4D, G). This became very important at P50 (Fig. 4G) suggesting strong metabolic reaction such as autophagy [22].

As melanin amount decreased in *Otx2* mutant retinas, we investigated expression of genes involved in its synthesis. Tyrosinase and DOPAchrome tautomerase (DCT) control two critical steps of melanogenesis, and Otx2 can transactivate both genes' promoter *in vitro* [7], [23]. We therefore tested the status of *Dct* and *tyrosinase* mRNA in mutants by RT-qPCR. Whereas *Dct* expression appeared unchanged (Fig. 4J), *tyrosinase* mRNA accumulation decreased (Fig. 4K), with kinetics that strictly paralleled the one of *Otx2* mRNA shown in Fig. 1A.

We conclude from these data that *Otx2* knockout in adult retina results in disruption of photoreceptor-RPE cell adhesion and in impaired melanogenesis in RPE cells. These defects as well as downregulation of tyrosinase start soon after knockout.

## Discussion

Our observations demonstrate that *Otx2* knockout induces profound alterations of RPE and photoreceptor cells, in adults. In our model, the most conspicuous retinal phenotype was the degeneration of photoreceptor cells. This process was very progressive, lasting for about 4 months following knockout, but ended in complete loss of photoreceptors. *Otx2* knockout also resulted in profound alterations of RPE such as reduction of melanosome size and number, which were evident at a much earlier time than most photoreceptor alterations.

Several studies have previously shown a role of *Otx2* in early or late development of RPE, retina, and in the differentiation of some retinal cell types [7], [8], [9], [10], [11], [14], [24]. Our study demonstrates an important role of this gene in adult retina. This is due to our use of a time-controlled Cre line, which allows *Otx2* invalidation, with very high efficiency, in all cells that express *Otx2*. To distinguish unambiguously defects that are cell-autonomous from those that result from cell-cell interactions, it will be necessary to trigger *Otx2* knockout only in restricted cell types in the adult. This will require other inducible Cre lines –e.g. for photoreceptor, bipolar, or RPE cells– or alternative experimental approaches. Several retinal cell-specific Cre lines have being made available, but most of them are not inducible or use promoters that are not efficiently expressed in adults. For instance, the RPE-specific Mct3-CreER^T2^ transgenic mouse described recently causes recombination only in 5% of RPE cells in adults [25]. On the other hand our technology provides for a quasi complete knockout of *Otx2*, which for some purposes (among which include a considerably greater ease in analyzing changes in gene expression induced by loss of Otx2 function) is advantageous.

From our study it seems clear that Otx2 plays a direct role in maintaining the integrity of RPE cells and in one of their major functions: melanogenesis. This is because knockout of *Otx2* results in a quick alteration of these cells, and reduction of their melanosome content. Such a reduction exposes mutant RPE cells to oxidative damages. As these induce an increase in intracellular vacuoles [26], they could explain the vacuolization we observe in RPE cells 20 days after *Otx2* gene invalidation. In addition we observe a synchronous decrease of *tyrosinase* and *Otx2* mRNA after tamoxifen treatment. In avian retina cells, Otx2 induces a pigmented phenotype and *in vitro*, the protein binds to a *bicoid* site present in the promoter of the *tyrosinase* gene [7]. This adds evidence in favour of *tyrosinase* as an Otx2 direct target gene *in vivo*, although direct proof of this will be required. Given the importance of RPE cells in nurturing and maintenance of photoreceptors, impairment of RPE functions could largely explain the slow photoreceptor degeneration that we observed. Melanosomes protect the retina from photo-oxidation that can be intense in RPE which lies against the oxygen rich choriocapillaris and receives focused light [27]. Another fast change in RPE cells is the widening of the subretinal space between RPE apical microvilli and photoreceptor outer segments. This might impair at least three RPE functions: trans-epithelial transport, visual cycle and phagocytosis, a condition that can lead to retinal degeneration [28]. It could involve adhesion molecules and extra-cellular matrix components. For instance, *Ezrin* mutants present reductions in the apical microvilli and basal infoldings in RPE cells [29]. Disruption of RPE-photoreceptor cell adhesion could also affect the homeostasis of the inter-photoreceptor matrix, which could explain the observed reduction of IRBP protein despite normal transcription of the *Irbp* gene.

Alternatively, or additionally, a slow cell-autonomous degeneration could be induced after *Otx2* knockout in photoreceptor cells, independent of RPE abnormalities. Although on the whole we detected severe disorders in photoreceptors following an appreciable lag compared to changes within RPE, some defects were noted in photoreceptors soon after *Otx2* knockout: 10 days after treatment there already appeared alterations in the outer segments of photoreceptors at their juncture with RPE (only visible with electron microscopy), and the loss of IRBP, likely reflecting a loosening of the matrix around photoreceptors, was already visible 4 days after tamoxifen treatment. Both changes however might be a consequence of defects autonomous to RPE cells, rather than to photoreceptors. It is remarkable that TUNEL staining detected apoptotic photoreceptor cells only 20 days following tamoxifen treatment. It is also possible, that our procedure resulted in incomplete knockout of *Otx2* in photoreceptors. As Otx2 seems to be expressed at a lower level there, the Cre recombinase might also be expressed at a low level which would decrease the fraction of cells in which it will act. We think this possibility unlikely because we observed a very high rate of recombination and a very strong reduction in Otx2 expression in all instances where we have used this strategy [18]. Moreover, we have noted before that in photoreceptor cells, Otx2 is concentrated in the periphery of the nucleus [12] – in other words, each cell could express a high level of Otx2 in a relatively small subcellular compartment.

It therefore seems plausible that the severe alterations in RPE cells, which express a high level of Otx2, caused the process of photoreceptor degeneration. At the same time we cannot exclude that disruption of Otx2 function might result in a delayed and staged schedule of apoptosis of these cells. This interesting possibility remains to be investigated - we note that in knockout mice for another homeoprotein, Engrailed, there occurs also a progressive loss of dopaminergic neurons [30]. The use of cell-specific inducible Cre mice will in any case allow us to address these issues in the future.

Although the main focus of this study was not to investigate which genes are downstream of Otx2, our results suggest that embryonic and adult Otx2 protein functions differ greatly. For instance although *Crx* expression is controlled by Otx2 in precursors of photoreceptor cells [31] we find that this is no longer the case in adult retina. This is of special interest as many retinal degenerative diseases are not developmental pathologies. They occur in adults and may be the consequence of somatic mutations or gene dosage in patients carrying heterozygous mutations [14]. It will be important to discover what are the targets of Otx2 in the mature retina and to look for *de novo OTX2* mutations in patients suffering specifically from late onset retinal diseases. The subset of *OTX2* mutations that should be expected would likely involve promoter or cis-regulatory change. Indeed, mutations affecting OTX2 protein coding sequence have early and severe effects on brain and eye development [14], [24]. Finding mutations outside the coding sequence may be difficult as regulatory elements of this small gene are scattered over large distances [16], [17]. However, we previously showed that *Otx2* gene is expressed from three different stage- and tissue-specific promoters, the second of them accounting for most of retinal transcripts in adults [15], [32]. Mutations affecting this promoter could therefore specifically reduce the amount of Otx2 protein in the retina without affecting brain and eye development.

## Methods

### Mouse breeding and tamoxifen administration

Generation of mouse lines (129Sv background) and genotyping were done as previously described [12], [18]. Mice were housed with a 12 h light/dark cycle. One intraperitoneal injection of tamoxifen (Sigma-Aldrich, St Louis, MO, USA) (10 mg/ml in sunflower oil) was done at 1 mg per 20 g body weight at 3:00 pm. All experiments were conducted under guidelines approved by local and state ethical committees. TL received the authorization to experiment #69101850 from the Veterinarian Service of the Préfecture du Rhône, France, and protocols used in this study were approved by the Comité Régional d'Ethique Rhône-Alpes (decision #0175).

### Immunocytochemistry

Eyes were fixed in 4% paraformaldehyde in phosphate-buffered saline (PBS) for 3 hours at 4°C, rinsed twice (30 min) in PBS, protected in PBS-sucrose (10–30%) and frozen in Tissue-Tek OCT at −80°C. Sections (8 µm) were mounted onto SuperFrost + slides (Fisher Scientific, Atlanta, GA), blocked 1 hour in PBT (PBS with 0.2% Gelatin and 0.1% Triton X-100) containing 5% donkey serum and incubated overnight at 4°C with the primary antibodies diluted in PBT with 2% donkey serum. After rinsing, slides were incubated 1 hour at 20°C with the secondary antibodies. Specimens were washed twice 15 min in PBS, coverslipped with Vectashield, observed under fluorescent Axioplan microscope (Carl Zeiss, Germany) and images analysed using *Metamorph* (Molecular Devices, Sunnyvale, CA, USA).

Antibodies and working dilutions. Primary antibodies: Monoclonal anti-RPE65 (1/500) (Abcam, Cambridge, UK), anti-syntaxin (1/1,000) (Sigma-Aldrich), anti-PKCalpha (1/1,000) (Santa Cruz Biotechnology; Santa Cruz CA, USA), anti-calbindin (1/500) (Sigma-Aldrich); Rabbit anti-rhodopsin (1/1,000) (Sigma-Aldrich); Goat anti-Brn-3 (1/100) and Goat anti-GFAP (1/200) (Santa Cruz Biotechnology); Rabbit anti-IRBP (1/200) (kind gift from B. Wiggert's lab); Goat anti-Otx2 (1/250) (R&D systems, Minneapolis, MN, USA) and Rat anti-Otx (1/200) (kind gift from M. Wassef).

Secondary antibodies (1/1,000): Donkey anti-Mouse Alexa Fluor 488, anti-Mouse Alexa Fluor 647, anti-Goat Alexa Fluor 647, anti-Rat Alexa Fluor 488 and anti-Rabbit Alexa Fluor 555 (Invitrogen, Carlsbad CA, USA). Cone staining used FITC-labeled peanut agglutinin (PNA, generous gift of O. Dkhissi-Benyahya).

### Histological studies and cell counting

Eyes were processed as above. Sections were stained with haematoxylin and eosin according to standard protocol. Cell numbers were determined by counting the nuclei in a 50 µm wide region of retinal section located at equal distance from the ora serrata and the optic disk. For each point, three eyes were dissected. For each, three different regions were counted. Average cell numbers and standard deviation were calculated using Statlab (SPSS Inc, Chicago, Illinois, USA). TUNEL assay was performed with the In Situ Cell Death Detection Kit, TMR red (Roche, Basel, Switzerland), according to the manufacturer's instructions.

### RNA Isolation and RT-qPCR

Retina were dissected at 3:00 pm in PBS. Total RNA was prepared with TRIzol (Invitrogen). First-strand cDNA was synthesized using 1 µg of total mRNA, MLV reverse transcriptase (Promega, Madison WI, USA) and random hexamers. For real time PCR, 1/100 of cDNA was used per reaction using PowerSYBR green PCR mix and StepOne Plus apparatus and software (Applied Biosystems, Foster City, CA, USA). Comparable efficiency of each PCR reaction was first demonstrated using serial dilutions of control cDNA. Gene to *TBP* ratios were determined from three independent assays by the 2^−DCt^ method [33]. PCR conditions and primers used: PCR cycles: 15 s at 95°C, 30 s at 60°C, 30 s at 72°C. Primers forward and reverse (amplicon size):

*Otx2* exon 1–2: 5′-ccaaatctacccaccaagga, 5′-agagcttccagaacgtcgag (276 bp)

*Otx2* exon 3: 5′-GCTGGCTCAACTTCCTACT, 5′-TCCAAGCAGTCAGCATTGAAG (260 bp)

*Tyrosinase*: 5′-ATTGATTTTGCCCATGAAGCA, 5′-TTCCATCGCATAAAACCTGAT (283 bp)

*Crx*: 5′-TGTATGCACGTGAGGAGGT, 5′-GCTGGACTCCAAATGGACA (301 bp)

*Irbp*: 5′-GGACCCACACTCAGCTCA, 5′-AGACAACTACATTAGGTGTCA (285 bp)

*Dct*: 5′-GCTACAATTACGCCGTTGAT, 5′-TTCCACCTGTCTCAAGATGA (268 bp)

*TBP*: 5′-TCTGGAAAAGTTGTATTAACAG, 5′-GCTGCAGGGTGATTTCAGT (284 bp)

*Gnat2: 5′-* TCTGTTTTCCGGAGTATGAC, 5′- TGCATGAAGCCTCAGATTCT (270 bp)

*Rho: 5′-* TGTGGCCTTCTACATCTTCA, 5′- ACAGGAGACTCCTACTTTCA (278 bp)

*Grk1: 5′-* AGCTGGAGGCAGCTCGAA, 5′- ACATTCCCGACTTGGCTGT (307 bp)

*Opn1: 5′-* CTACTGCTTCATGAATAAGCA, 5′- GAAGGTGACTCACCAGAGT (266 bp)

*Pde6b: 5′-* CGATTTCACGAAGAGATCCT, 5′- TAGGCAGAGTCCGTATGCA (271 bp)

### Western blot

Retinas were dissected in cold PBS and nuclear protein extracts [34] were prepared as described previously [35]. Proteins (30 µg) were fractionated on 10% SDS-PAGE and transferred onto nitrocellulose (Optitrans, Schleicher & Schuell, Dassel, Germany) and incubated with rat anti-Otx antibody (1/1,000). Revelation was done using HRP-coupled goat anti-rat antibody (1/10,000) (Jackson ImmunoResearch, West Grove, PA, USA).

### Transmission electron microscopy (TEM)

Mice were perfused transcardially under deep anaesthesia with PBS, then fixative (1% paraformaldehyde, 3% glutaraldehyde in phosphate buffer 0.1 M, pH 7,4). Eyes were fixed 2 h at 4°C. Corneas were removed and eyes fixed 2 h. Lens were removed and eyecups fixed 2 h. Ten 1×1 mm retinal strips were processed separately. Samples were osmicated, dehydrated with ethanol series. Each sample was embedded in Epon mixture and 0.5 µm thin sections were stained with toluidine blue for light microscopy followed by ultra thin section preparation for TEM (Philips CM120, Eindhoven, NL).

## Supporting Information

Figure S1 Otx2 protein in adult retina. Expression of Otx2-GFP protein in adult (P60) retina of *Otx2^+/Otx2−GFP^* mouse. Direct protein fluorescence (green) of a vertical section is shown. RPE: retinal pigment epithelium; ONL: outer nuclear layer; INL: inner nuclear layer; GCL: ganglion cell layer.
(0.64 MB TIF)

Figure S2 The self knockout strategy. The structure of alleles used for self knockout is presented at the top with *Otx2* exons as boxes (gray: non coding, light blue: coding regions, dark blue: homeobox), knock-in *CreERT2* (yellow box) and *neo* (blue box) genes and *loxP* sites (red triangles). Black arrows are transcription start sites. PCR primer pairs are blue and purple arrows. The structure resulting from tamoxifen-induced CreERT2 activity is shown at the bottom.
(0.26 MB TIF)

Figure S3 *Otx2* driven *CreERT2* expression is stable in the absence of Otx2. In situ hybridization was performed 30 days post injection on retina of control or tamoxifen injected *Otx2^flox/CreERT2^* mice using the whole CreERT2 coding sequence as a probe. RPE: retinal pigment epithelium; ONL: outer nuclear layer; INL: inner nuclear layer; GCL: ganglion cell layer.
(0.71 MB TIF)

Figure S4 Absence of tamoxifen toxicity. Histology of P160 control and treated (Tamoxifen administrated at P30) retinas of the indicated genotypes. Sections are stained with Eosin and Haematoxylin. RPE: retinal pigment epithelium; ONL: outer nuclear layer; INL: inner nuclear layer; GCL: ganglion cell layer.
(0.80 MB TIF)

Figure S5 *Crx* expression is independent of Otx2 in adult retina. In situ hybridization was performed on P60 sections of control and mutant retina after tamoxifen treatment at P30 with full-length *Crx* cDNA (a kind gift of C. Cepko) as a probe. RPE: retinal pigment epithelium; ONL: outer nuclear layer; INL: inner nuclear layer; GCL: ganglion cell layer.
(0.44 MB TIF)

Figure S6 Stable expression of several photoreceptor markers following *Otx2* self-knockout. RT-qPCR quantization of mRNA abundance was performed with the indicated photoreceptor specific genes. Shown is the mean of three independent experiments. Error bars are standard deviation.
(0.36 MB TIF)
