# Supplementary figures and images for: Otx2 Gene Deletion in Adult Mouse Retina Induces Rapid RPE Dystrophy and Slow Photoreceptor Degeneration
Source: PLoS One. 2010 Jul 21;5(7):e11673. doi: 10.1371/journal.pone.0011673 (PMC2908139; doi:10.1371/journal.pone.0011673)

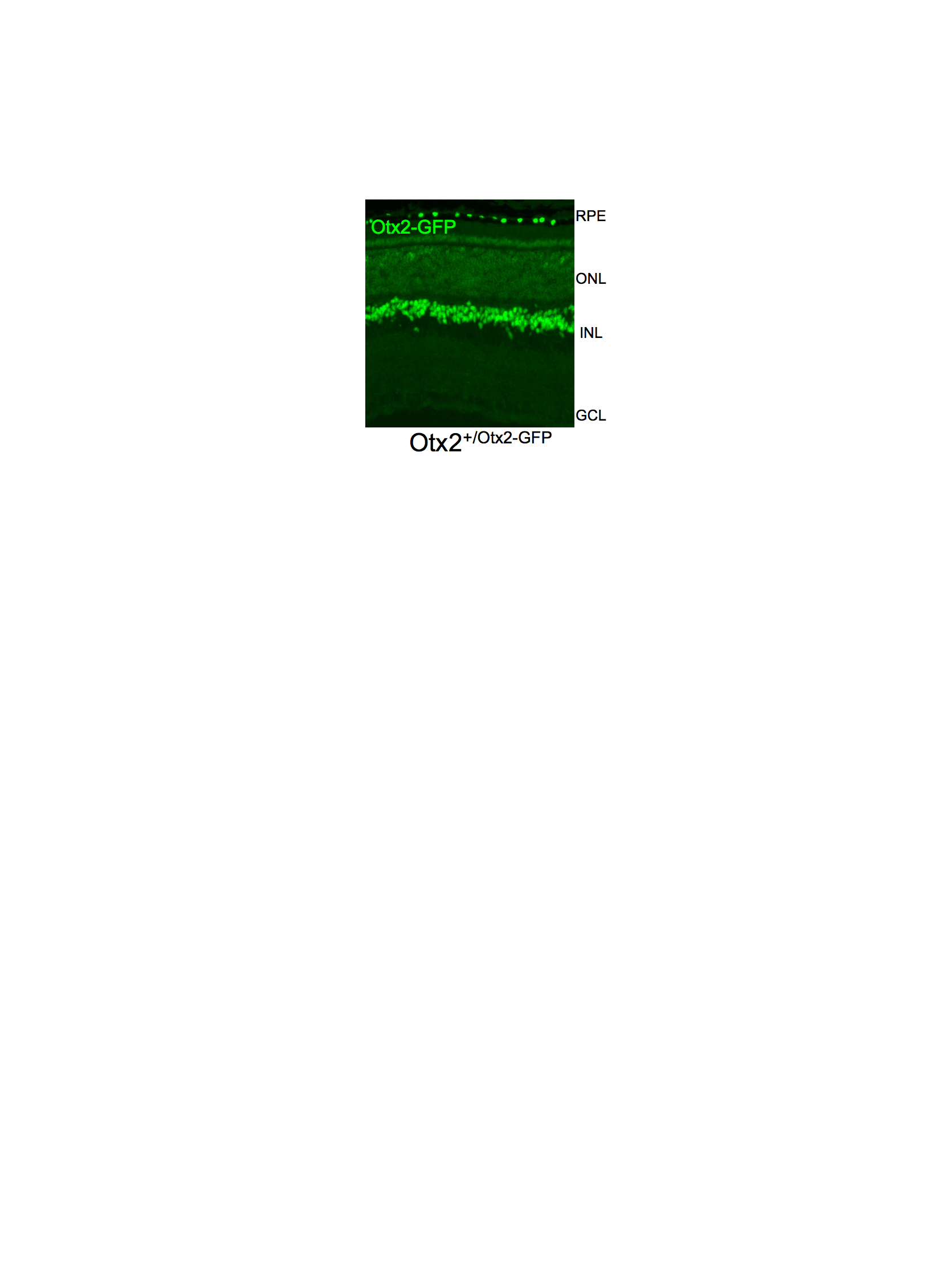

Supplement: Figure S1 — Otx2 protein in adult retina. Expression of Otx2-GFP protein in adult (P60) retina of Otx2+/Otx2−GFP mouse. Direct protein fluorescence (green) of a vertical section is shown. RPE: retinal pigment epithelium; ONL: outer nuclear layer; INL: inner nuclear layer; GCL: ganglion cell layer. (0.64 MB TIF) [file pone.0011673.s001.tif]

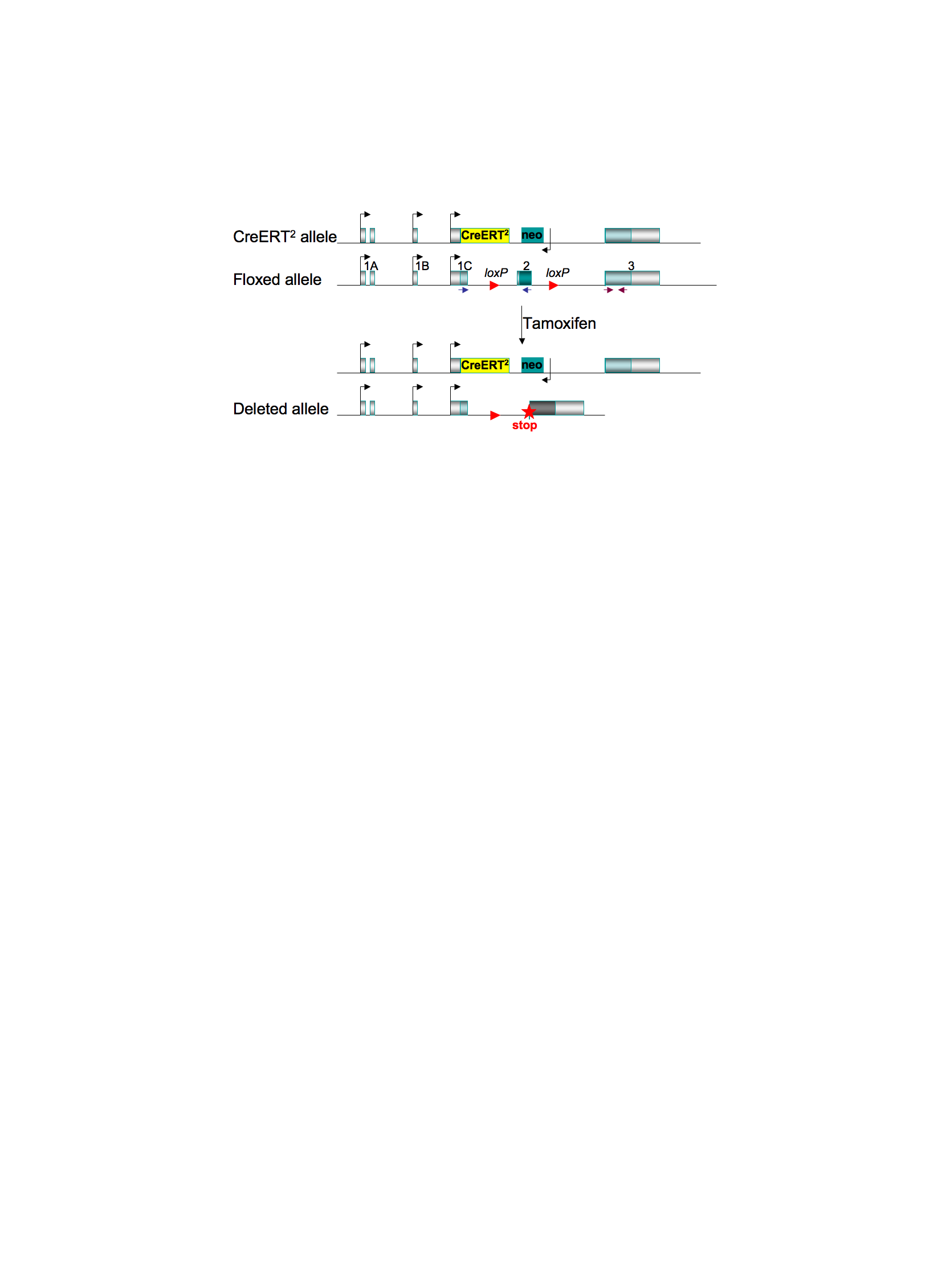

Supplement: Figure S2 — The self knockout strategy. The structure of alleles used for self knockout is presented at the top with Otx2 exons as boxes (gray: non coding, light blue: coding regions, dark blue: homeobox), knock-in CreERT2 (yellow box) and neo (blue box) genes and loxP sites (red triangles). Black arrows are transcription start sites. PCR primer pairs are blue and purple arrows. The structure resulting from tamoxifen-induced CreERT2 activity is shown at the bottom. (0.26 MB TIF) [file pone.0011673.s002.tif]

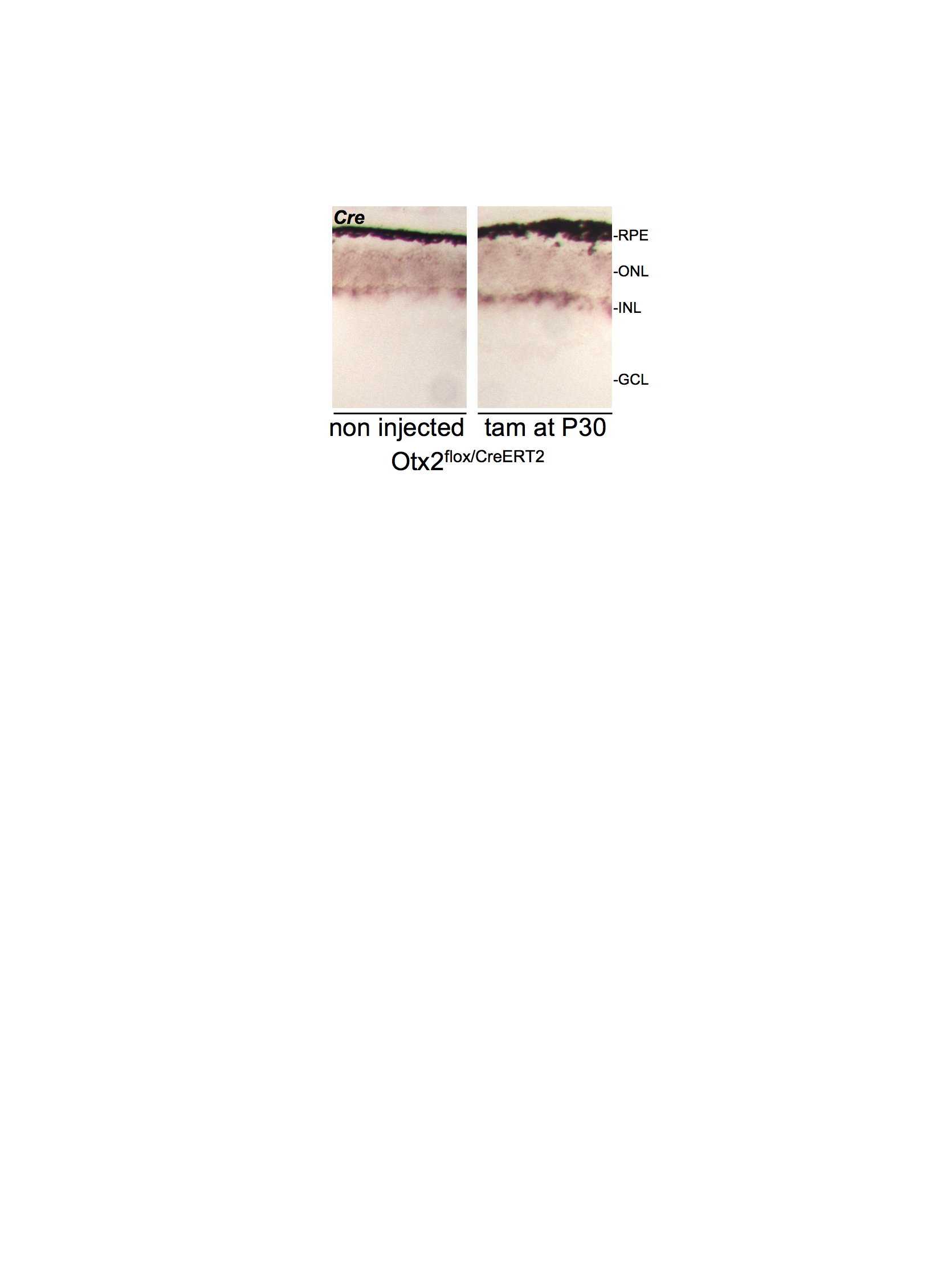

Supplement: Figure S3 — Otx2 driven CreERT2 expression is stable in the absence of Otx2. In situ hybridization was performed 30 days post injection on retina of control or tamoxifen injected Otx2flox/CreERT2 mice using the whole CreERT2 coding sequence as a probe. RPE: retinal pigment epithelium; ONL: outer nuclear layer; INL: inner nuclear layer; GCL: ganglion cell layer. (0.71 MB TIF) [file pone.0011673.s003.tif]

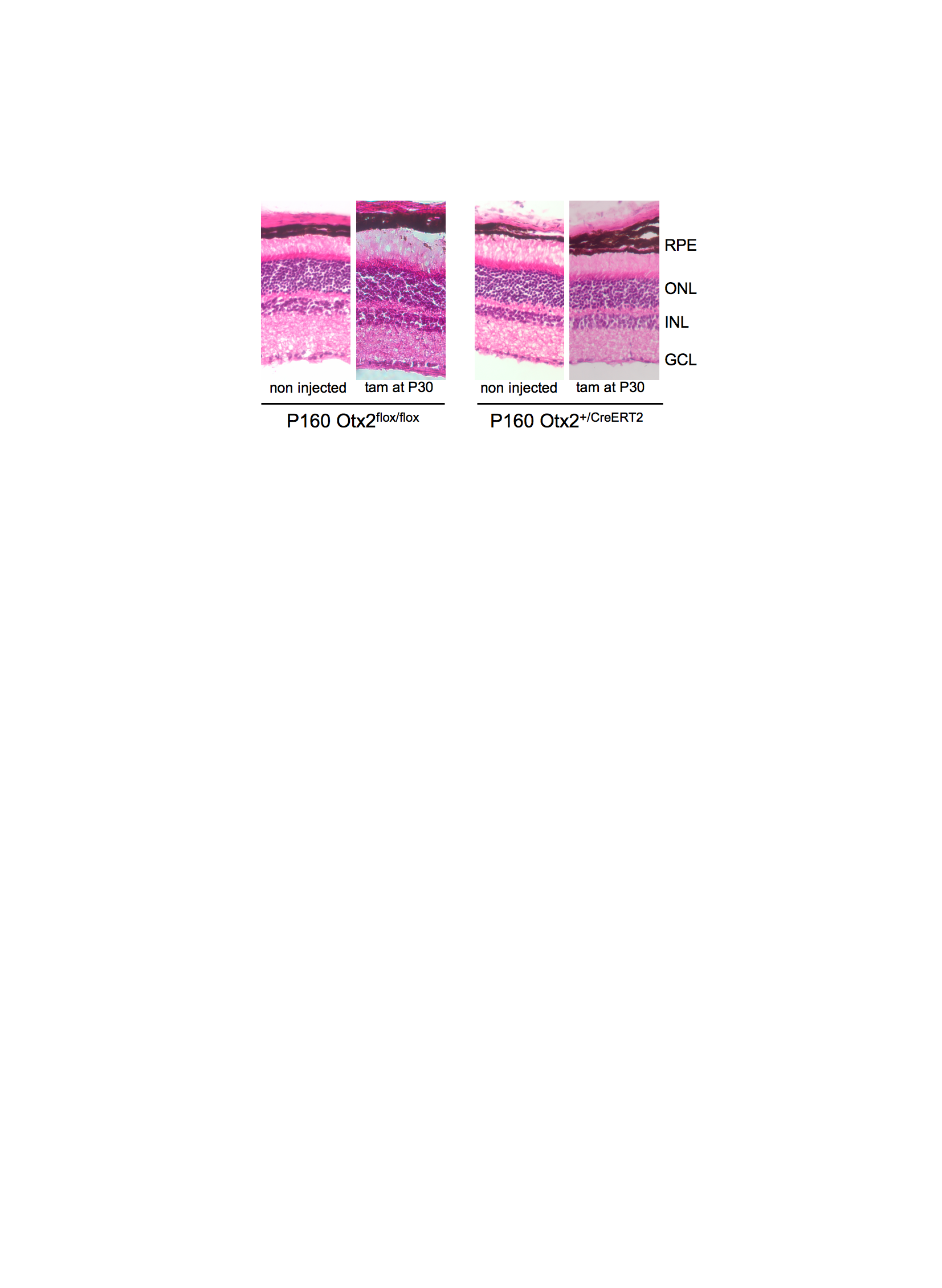

Supplement: Figure S4 — Absence of tamoxifen toxicity. Histology of P160 control and treated (Tamoxifen administrated at P30) retinas of the indicated genotypes. Sections are stained with Eosin and Haematoxylin. RPE: retinal pigment epithelium; ONL: outer nuclear layer; INL: inner nuclear layer; GCL: ganglion cell layer. (0.80 MB TIF) [file pone.0011673.s004.tif]

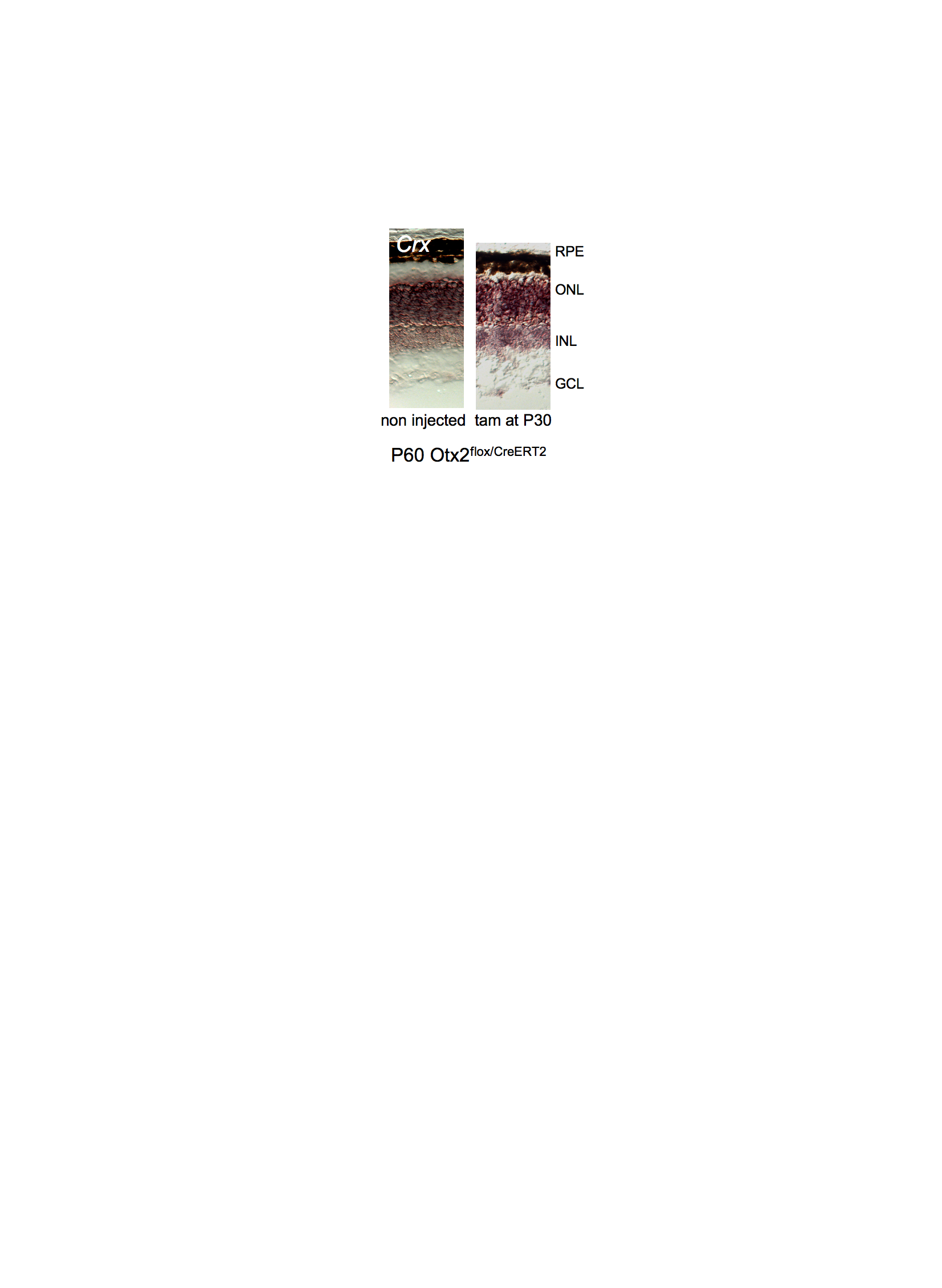

Supplement: Figure S5 — Crx expression is independent of Otx2 in adult retina. In situ hybridization was performed on P60 sections of control and mutant retina after tamoxifen treatment at P30 with full-length Crx cDNA (a kind gift of C. Cepko) as a probe. RPE: retinal pigment epithelium; ONL: outer nuclear layer; INL: inner nuclear layer; GCL: ganglion cell layer. (0.44 MB TIF) [file pone.0011673.s005.tif]

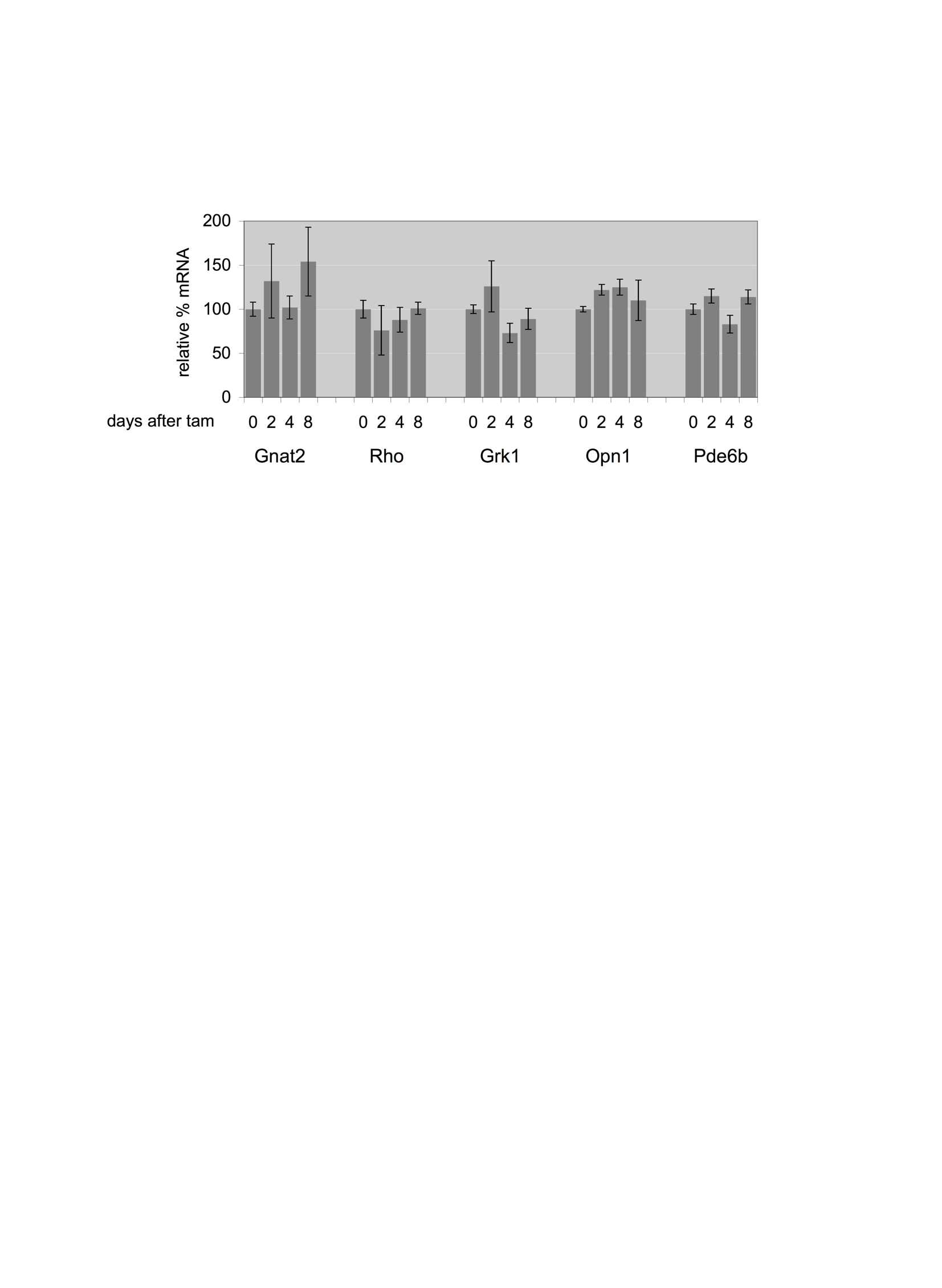

Supplement: Figure S6 — Stable expression of several photoreceptor markers following Otx2 self-knockout. RT-qPCR quantization of mRNA abundance was performed with the indicated photoreceptor specific genes. Shown is the mean of three independent experiments. Error bars are standard deviation. (0.36 MB TIF) [file pone.0011673.s006.tif]
